# Radiographic Number of Positive Pelvic Lymph Nodes as a Prognostic Factor in Cervical Cancer Treated With Definitive Concurrent Chemoradiotherapy or Intensity-Modulated Radiotherapy

**DOI:** 10.3389/fonc.2018.00546

**Published:** 2018-11-30

**Authors:** Shih-Chang Wang, Li-Ching Lin, Yu-Ting Kuo, Yu-Wei Lin

**Affiliations:** ^1^Department of Radiation Oncology, Chi-Mei Medical Center, Tainan, Taiwan; ^2^Department of Medical Imaging, Chi-Mei Medical Center, Tainan, Taiwan

**Keywords:** cervical cancer, positive lymph nodes, radiographic finding, survival, CCRT, IMRT, pelvic lymph node

## Abstract

**Background:** This study aims to assess the prognostic significance of radiographic numbers of positive pelvic lymph nodes (PLNs) in patients with cervical cancer treated with definitive concurrent chemoradiotherapy (CCRT) or intensity-modulated radiotherapy (IMRT).

**Methods:** We conducted a retrospective study that included 164 eligible adult patients with cervical cancer who were treated with definitive CCRT or IMRT at our institution from 2009 to 2016. After exclusion of 50 patients, a total of 114 patients whose clinicopathological data and follow-up were finally analyzed. The radiographic numbers of positive PLNs were assessed by pretreatment magnetic resonance imaging (MRI) or computed tomography (CT). The criterion for a positive lymph node was defined as a short-axis diameter >1 cm. Using the Kaplan–Meier method and the Cox proportional hazards regression model, we assessed the overall survival (OS), cancer-specific survival (CSS), distant metastasis-free survival (DMFS), and locoregional relapse-free survival (LRFS).

**Results:** The median follow-up duration was 40 (range: 2–100) months. For patients with 0, 1–2, and ≥3 positive PLNs, the estimated 3-year OS were 85.4% vs. 82.4% vs. 59.7% (*p* = 0.035), CSS were 90.1% vs. 86.1% vs. 62.9% (*p* = 0.010), DMFS were 89.4% vs. 91.3% vs. 49.6% (*p* < 0.001), and LRFS were 77.8% vs. 73.4% vs. 70% (*p* = 0.690). Per the multivariate Cox regression, positive PLNs ≥3 (HR, 2.51; 95% CI: 1.09–5.80; *p* = 0.031) and non-squamous cell carcinoma type (HR, 2.82; 95% CI: 1.19–6.69; *p* = 0.018) were unfavorable factors for the OS. Besides, positive PLNs ≥3 was the independent factor for the CSS (HR, 3.38; 95% CI: 1.32–8.67; *p* = 0.011) and DMFS (HR, 6.83; 95% CI: 2.62–17.83; *p* < 0.001). The patients that were treated without intracavitary brachytherapy exhibited inferior LRFS (HR, 13.15; 95% CI: 2.66–65.10; *p* = 0.002).

**Conclusions:** The radiographic number of positive PLNs (≥ 3) is an independent prognostic factor for OS, CSS, and DMFS in patients treated with definitive CCRT or IMRT.

## Introduction

Cervical cancer is the fourth leading malignancy among females worldwide (1). In 2015, 526,000 females were diagnosed with cervical cancer worldwide, accounting for 239,000 deaths (2). The standard treatment for early-stage cervical cancer is radical surgery or definitive radiotherapy (RT). However, for locally advanced cervical cancer, concurrent chemotherapy with radiotherapy (CCRT) is considered the main treatment (3). Lately, intensity-modulated radiotherapy (IMRT), an advanced radiotherapy technique, has been proven to decrease the gastrointestinal toxicity and can be used to increase the radiotherapy dose selectively (4, 5). Despite remarkable advances in the treatment of cervical cancer, disease failure and mortality are still prevalent in patients. Research has revealed that the rates of locoregional recurrence and distant metastasis in patients with cervical cancer treated with definitive IMRT are 5–23% and 11–35%, respectively (6–9). Patients who experience a local relapse of disease could be eligible for salvage surgery; however, the treatment for patients with distant metastases is challenging and is associated with poor survival outcomes.

In cervical cancer, the tumor size, histological subtype, baseline hemoglobin, and involvement of pelvic or para-aortic lymph nodes have been identified to possess a prognostic value (10–14). Some studies have suggested that the lymph node positivity is one of the most prominent prognostic factors for recurrence and death in patients with cervical cancer (15, 16). Conversely, other studies reported conflicting results that positive pelvic lymph nodes (PLNs) do not affect recurrence and survival (17, 18). A GOG study about early-stage cervical cancer undergoing surgery showed significantly worse disease free interval between patients with positive pelvic lymph nodes but not survival (19). Although there is some evidence regarding the prognostic significance of numbers of involved lymph nodes in women who have undergone surgery for early stage cervical cancers (20, 21), there are limited data regarding the prognostic significance of the numbers of involved nodes in women with locally advanced cervical cancer. Therefore, this study aims to evaluate the prognostic factors in patients with advanced cervical cancer who treated with definitive CCRT or IMRT.

## Methods and materials

### Patients

The patients in this retrospective study were identified from a database of patients who were diagnosed with cervical cancer in Chi-Mei Medical Center between 2009 and 2016. The study was approved by our institutional review board (10608-005). The eligibility criteria were as follows: (1) patients with pathologically confirmed cervical cancer who were >18 year's old; (2) patients who underwent definitive CCRT or IMRT, without prior radical surgery. The exclusion criteria were as follows: (1) recurrent disease; (2) distant metastasis other than para-aortic lymph node at diagnosis; (3) incomplete the planned CCRT or RT; (4) missing data. The pretreatment workup comprised patients' history, physical and gynecological examination, laboratory tests, and image studies. The clinical stage was determined on the basis of a multidisciplinary consensus between gynecological oncologist, radiologist, and radiation oncologist.

Abdominal-pelvic MRI or CT was performed in all eligible patients, but PET-CT was only utilized in only 7 eligible patients. Therefore, the evaluation of the lymph node involvement was based on MRI or CT radiographic findings. The principal criteria for the positive node involvement were based on the axial diameter of the lymph node. Notably, lymph nodes >1 cm in the short-axis dimension were considered abnormal. We defined the positive PLN as an abnormal lymph node located at the common iliac, external iliac, internal iliac, obturator, and presacral area. And the para-aortic region is the lymphatic region along aorta and inferior vena cava in between the renal hilum to the common iliac bifurcation. All the MRI or CT images were concurrently reviewed and evaluated independently by two qualified doctors without any prior knowledge of each patient's clinical details. When the opinions of the two evaluators differed, consensus was reached by discussion.

### Treatment

All the patients included in this study underwent radiotherapy comprised external beam radiotherapy (EBRT) using IMRT technique with (without) high-dose-rate intracavitary brachytherapy (ICBT). EBRT was delivered to the whole pelvis using 6- or 10-MV photons in 1.8–2.0 Gray (Gy) daily fractions, five times a week. While the high-risk clinical target volume (CTV) covered the cervical tumor, parametrium, and gross lymph nodes, the low-risk CTV covered the entire pelvis area with or without the para-aortic region. The median EBRT dose to high-risk CTV was 50.4 Gy, and the median dose to low-risk CTV was 45 Gy. All except 12 patients received high-dose-rate ICBT using the remote afterloading system with the iridium-192 source. In addition, brachytherapy was delivered two times a week with the median dose of 25 (range: 13.5–30) Gy to point A in three to six fractions. The total equivalent doses in 2-Gy fractions (EQD2) were 45.0–96.1 (median: 81.2) Gy. Furthermore, chemotherapy comprised weekly cisplatin (40 mg/m^2^) that was delivered concurrently with EBRT.

### Follow-up

After the treatment completion, patients were examined every 3 months for the first year and every 6 months after that. We defined a clinical complete response as no evidence of disease 3 months after the treatment completion based on MRI or CT imaging studies. At the time of each evaluation, the toxicity was assessed per the National Cancer Institute Common Terminology Criteria for Adverse (CTCAE) v3.0. In addition, the overall survival (OS), cancer-specific survival (CSS), distant metastasis-free survival (DMFS), and locoregional relapse-free survival (LRFS) were evaluated from the date of diagnosis to the date of the event, and the surviving patients were censored on the date of the last follow-up.

### Statistical analysis

While the Kaplan–Meier method was used to construct the survival curves, the log-rank test was used to compare the differences in the survival rates. The Cox proportional hazards regression model was used to assess the prognostic factors to estimate survival outcomes. We considered *P* < 0.05 as statistically significant. SPSS version 19.0 (SPSS Inc., Chicago, IL) was used to perform all the statistical analyses.

## Results

### Patient and treatment characteristics

A total of 164 patients were initially eligible. Fifty patients were excluded due to recurrent disease (*n* = 12); distant metastasis other than para-aortic lymph node at diagnosis (*n* = 22); incomplete the planned CCRT or RT (*n* = 8); and missing data (*n* = 8). After exclusion of 50 patients, a total of 114 patients whose clinicopathological data and follow-up were finally analyzed. Table 1 summarizes the clinicopathological characteristics of the study cohort. Based on the American Joint Committee on Cancer (AJCC) staging, 18 (16%), 38 (33%), 36 (32%), and 22 (19%) of the patients had stage I, II, III, and IV cervical cancer. Of the 114 patients, 94 (82%) had squamous cell carcinoma, 16 (14%) had adenocarcinoma, and 4 (4%) had poorly differentiated, undifferentiated, or adenosquamous carcinoma. We observed the involvement of para-aortic in 11 (10%) patients. 69 (61%), 24 (21%), and 21(18%) of the patients had the involvement of 0, 1–2, ≥3 PLNs, respectively. Most of the patients were treated with CCRT (95 cases, 83%), and ICBT was used in 102 patients (89%) after EBRT. The complete clinical and image response was attained in 82% of the patients. The treatment failure occurred in 40 (35%) patients, including locoregional failure alone (21 cases, 18%), distant failure alone (14 cases, 12%), and synchronous locoregional and distant failure (5 cases, 4%).

**Table 1:** Patient and treatment characteristics (*N* = 114).

**Characteristics**		**Number of patients (%)**
Age (years)	< 60	64 (56%)
	≧ 60	50 (44%)
	Median (range)	58 (31–92)
Initial stage	I	18 (16%)
	II	38 (33%)
	III	36 (32%)
	IV	22 (19%)
Histology	Squamous cell carcinoma	94 (82%)
	Adenocarcinoma	16 (14%)
	Others	4 (4%)
Para-aortic lymph node status	Uninvolved	103 (90%)
	Involved	11 (10%)
Pelvic Lymph node status	Uninvolved	69 (61%)
	1–2	24 (21%)
	≧ 3	21 (18%)
Concurrent chemotherapy	Yes	95 (83%)
	No	19 (17%)
EBRT dose (cGy)	4,500–5,400	101 (89%)
	>5,400	13 (11%)
	Median (range)	5,040 (4,500–7,160)
Intracavitary brachytherapy	Yes	102 (89%)
	No	12 (11%)
Point A EQD2 dose (cGy)	< 7,000	12 (11%)
	7,000–7,999	39 (34%)
	≧8,000	63 (55%)
	Median (range)	8,122 (4,500–9,609)
Pretreament Hb	< 10	36 (32%)
	≧10	78 (68%)
Clinical complete response	Yes	93 (82%)
	No	21 (18%)
Location of first recurrence	None	74 (65%)
	Locoregional alone	21 (18%)
	Distant alone	14 (12%)
	Both locoregional and distant	5 (4%)

*EBRT, external beam radiation therapy; EQD2, equivalent dose in 2 Gy fractions; Hb, hemoglobin*.

### Impact of radiographic number of positive lymph nodes on survival

**Figure 1:**
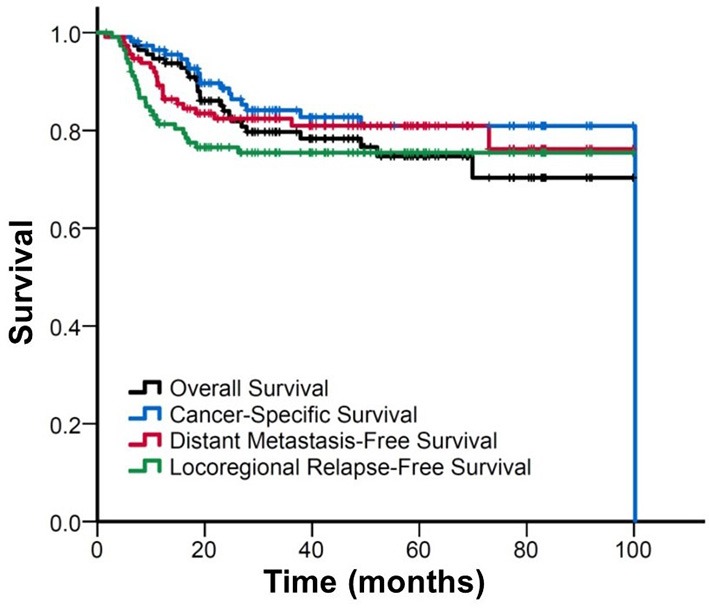
FigureKaplan–Meier curve of overall survival, cancer-specific survival, distant metastasis-free survival, and locoregional relapse-free survival for all patients.

The median follow-up duration was 40 (range: 2–100) months. Figure 1 shows the Kaplan–Meier curves for the survival. The estimated OS, CSS, DMFS, and LRFS rate were 79.9, 84.1, 82.4, and 75.5% at 3 years, and 75, 80.9, 81.0, and 75.5% at 5 years, respectively.

**Figure 2:**
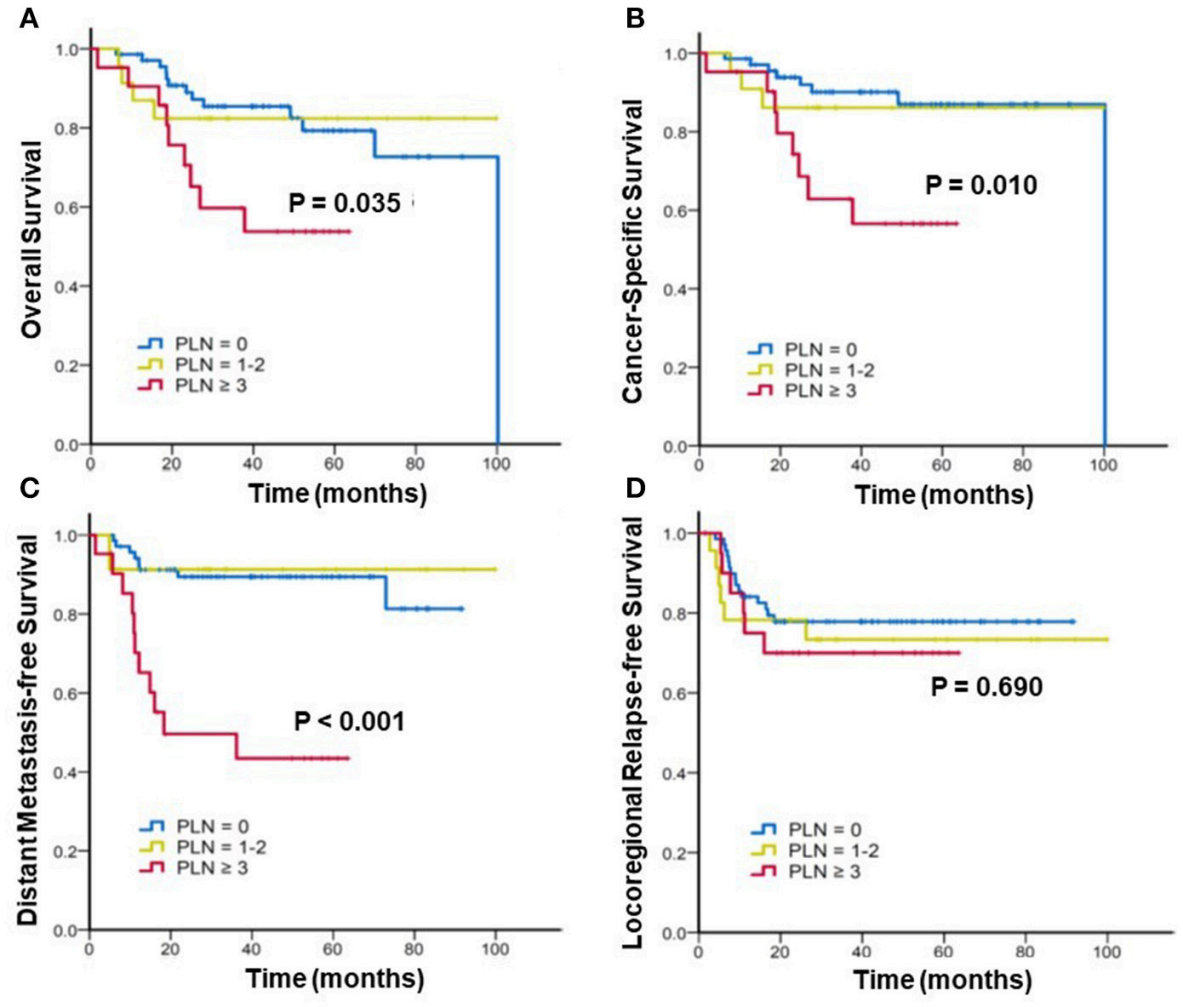
Kaplan–Meier analysis of **(A)** overall survival, **(B)** cancer-specific survival, **(C)** distant metastasis-free survival, and **(D)** locoregional relapse-free survival for patients with cervical cancer stratified by number(s) of positive pelvic lymph nodes (PLNs).

Furthermore, we stratified the patients into three subgroups to assess the effect of the radiographic number of positive PLNs on the survival. Figure 2 shows the Kaplan–Meier curves of the OS, CSS, DMFS, and LRFS for patients with 0, 1–2, and ≥3 positive PLNs. For patients with 0, 1–2, and ≥3 positive PLNs, the estimated 3-year OS were 85.4% vs. 82.4% vs. 59.7% (*p* = 0.035), CSS were 90.1% vs. 86.1% vs. 62.9% (*p* = 0.010), and DMFS were 89.4%, 91.3%, and 49.6% (*p* < 0.001). In patients with ≥3 positive PLNs, the DMFS, CSS, and OS were considerably worse. No significant difference was noted in the 3-year LRFS among the three subgroups (77.8% vs. 73.4% vs. 70%; *p* = 0.690).

### Cox regression analysis of the prognostic factors

Table 2 presents the results of the univariate and multivariate analyses. The multivariate analysis revealed that positive PLNs ≥3 (HR, 2.51; 95% CI: 1.09–5.80; *p* = 0.031) and non-squamous cell carcinoma type (HR, 2.82; 95% CI: 1.19–6.69; *p* = 0.018) exhibited poorer OS. Positive PLNs ≥3 was determined as the independent factor for the CSS (HR, 3.38; 95% CI: 1.32–8.67; *p* = 0.011) and DMFS (HR, 6.83; 95% CI: 2.62–17.83; *p* < 0.001) by the multivariate analysis. Furthermore, a significantly poor LRFS was observed in patients without ICBT compared with those with ICBT (HR, 13.15; 95% CI: 2.66–65.10; *p* = 0.002).

**Table 2:** Cox regression analysis of overall survival (OS), cancer-specific survival (CSS), distant metastasis-free survival (DMFS), locoregional relapse-free survival (LRFS).

	**OS**				**CSS**				**DMFS**				**LRFS**			
**Characteristics**	**Univariate**	**Multivariate**			**Univariate**	**Multivariate**			**Univariate**	**Multivariate**			**Univariate**	**Multivariate**		
	***p*-value**	**HR**	**(95% CI)**	***p*-value**	***p*-value**	**HR**	**(95% CI)**	***p*-value**	***p*-value**	**HR**	**(95% CI)**	***p*-value**	***p*-value**	**HR**	**(95% CI)**	***p*-value**
**AGE**																
≧60 vs. < 60	0.291	-	-	-	0.803	-	-	-	0.341	-	-	-	0.375	-	-	-
**INITIAL STAGE**																
IV vs. III vs. II vs. I	0.203	-	-	-	0.666	-	-	-	0.121	-	-	-	0.284	-	-	-
**HISTOLOGY**																
Non-SqCC vs. SqCC	0.007	2.82	(1.19–6.69)	0.018	0.021	2.82	(0.99–7.21)	0.053	0.163	-	-	-	0.133	-	-	-
**PALN**																
Involved vs. uninvolved	0.619	-	-	-	0.788	-	-	-	0.057	0.95	(0.29–3.12)	0.936	0.762	-	-	-
**PLN**																
Involved vs. uninvolved	0.168	-	-	-	0.055	-	-	-	0.021	-	-	-	0.466	-	-	-
**NUMBERS OF PLNs**																
≧3 vs. < 3	0.013	2.51	(1.09–5.80)	0.031	0.005	3.38	(1.32–8.67)	0.011	< 0.001	6.83	(2.62–17.83)	< 0.001	0.528	-	-	-
**CONCURRENT CHEMOTHERAPY**																
No vs. Yes	0.630	-	-	-	0.537	-	-	-	0.158	-	-	-	0.058	0.41	(0.08–1.98)	0.266
**INTRACAVITARY BRACHYTHERAPY**																
No vs. Yes	0.173	-	-	-	0.220	-	-	-	0.460	-	-	-	< 0.001	13.15	(2.66–65.10)	0.002
**POINT A EQD2 DOSE (cGy)**																
< 8,000 vs. ≧8,000	0.826	-	-	-	0.937	-	-	-	0.717	-	-	-	0.369	-	-	-
**PRETREATMENT Hb**																
< 10 vs. ≧10	0.267	-	-	-	0.773	-	-	-	0.753	-	-	-	0.725	-	-	-

*SqCC, Squamous cell carcinoma; PALN, para-aortic lymph node; PLN, pelvic lymph node; EQD2, equivalent dose in 2 Gy fractions; Hb, hemoglobin*.

In order to further clarify the prognostic role of positive PLNs, the data was re-analyzed by excluding the patients without brachytherapy which was the main factor for LRFS. The variant of chemotherapy was also put into multivariate analysis, because patient who did not receive chemotherapy may also contributed to the poorer outcome in patients with PLNs. Table 3 revealed that positive PLNs ≥3 was still associated with DMFS (HR, 5.28; 95% CI: 1.91–14.55; *p* = 0.001), and contributed to poor CSS (HR, 3.80; 95% CI: 1.24–11.66; *p* = 0.020), and OS (HR, 3.04; 95% CI: 1.06–8.72; *p* = 0.039) after excluding the patients without brachytherapy.

**Table 3:** Multi-variate Cox regression analysis of overall survival (OS), cancer-specific survival (CSS), distant metastasis-free survival (DMFS) by exclusion of patients who did not received brachytherapy.

	**OS**			**CSS**			**DMFS**		
**Characteristics**	**HR**	**(95% CI)**	***p*-value**	**HR**	**(95% CI)**	***p*-value**	**HR**	**(95% CI)**	***p*-value**
**HISTOLOGY**									
Non-SqCC vs. SqCC	2.57	(0.96–6.85)	0.060	2.38	(0.74–7.67)	0.146	1.31	(0.42–4.07)	0.643
**PALN**									
Involved vs. uninvolved	0.45	(0.09–2.25)	0.331	0.24	(0.03–2.03)	0.190	1.23	(0.36–4.12)	0.744
**NUMBERS OF PLNs**									
≧3 vs. < 3	3.04	(1.06–8.72)	0.039	3.80	(1.24–11.66)	0.020	5.28	(1.91–14.55)	0.001
**CONCURRENT CHEMOTHERAPY**									
No vs. Yes	1.85	(0.51–6.75)	0.455	0.84	(0.10–6.78)	0.867	0.87	(0.11–7.02)	0.897

*SqCC, Squamous cell carcinoma; PALN, para-aortic lymph node; PLN, pelvic lymph node*.

### Toxicities

In this study, no treatment-related deaths were reported. Table 4 presents acute and late toxicity of 114 patients. Only 1 patient (1%) experienced acute grade 3 gastrointestinal (GI) toxicity, and no grade 3 genitourinary (GU) toxicity was observed; this patient suffered from severe diarrhea (an increase of ≥7 stools/day over the baseline) during CCRT. In addition, only 1 patient who had stage IV cancer with tumor invasion to the bladder developed late grade 3 GU toxicity. Furthermore, right ureteral stricture and post-radiation ischemia bladder mucosa were diagnosed at 6 months after the initiation of radiotherapy, and operative intervention was performed.

**Table 4:** Radiation-related toxicity.

		**Grade 0–1**	**Grade 2**	**Grade 3**	**Grade 4**
Acute	GI	75 (66%)	38 (33%)	1 (1%)	0 (0%)
	GU	102 (89%)	12 (11%)	0 (0%)	0 (0%)
Late	GI	109 (96%)	5 (4%)	0 (0%)	0 (0%)
	GU	108 (95%)	5 (4%)	1 (1%)	0 (0%)

*Values are number of patients (%). GI, Gastrointestinal; GU, genitourinary*.

## Discussion

This study revealed that the number of positive PLNs ≥3 was an independent prognostic factor for the estimation of the OS, CSS, and DMFS in patients with cervical cancer treated with definitive CCRT or IMRT. In particular, this study focused on the prognostic factors for patients receiving non-surgical management. The number of positive PLNs in this study was assessed by MRI or CT radiographic finding.

Cervical cancer is a common gynecological malignant disease, typically characterized by lymph nodes metastases. The presence of PLN metastases has been related to the increased pelvic recurrence and distance metastases and a decline in the OS (22). However, the tumor–node–metastasis (TNM) staging system for cervical cancer based on the AJCC only considers whether patients have negative or positive lymph node (23). In addition, the lymph node status is not included in the International Federation of Gynecology and Obstetrics staging system (23, 24). Recently, the number of positive lymph nodes is assessed during lymph node staging in various malignant tumors, including breast cancer, esophageal cancer, colorectal cancer, and gastric cancer. In addition, the number of positive PLNs has been shown to correlate with prognosis and clinical outcomes in cervical cancer. Monk et al. and Hosaka et al. demonstrated that a higher number of positive lymph nodes correlated with adverse survival outcomes. The 5-year OS rate of patients with was 93–81% for one positive node, 77–66% for two nodes, and 33–31% for three nodes. The OS rate of patients with one or two lymph node(s) metastatic sites was considerably better than that for patients with more than two lymph nodes metastatic sites (20, 25). The multivariable Cox analysis in a comprehensive study with 2,222 patients revealed that patients with more than two positive lymph nodes exhibited poorer CSS (HR, 1.631; 95% CI: 1.382–1.926; *p* < 0.001) and OS (HR, 1.570; 95% CI: 1.346–1.832; *p* < 0.001) than patients with one or two positive lymph node(s) (21). Furthermore, the number of positive lymph nodes described above was verified by pathological findings after hysterectomy and lymphadenectomy.

Notably, the prognostic value of radiographic determination of lymph node metastases is crucial because a large portion of patients with cervical cancer do not receive surgical management (26). Liang et al. reported that the 4-year DFS for patients with cervical cancer with and without PLN metastasis assessed by MRI were 27 and 81%, respectively (27). Likewise, Daisuko et al. demonstrated by MRI that 29% of patients exhibited the PLN enlargement and were associated with the poor OS (28). Conversely, Wataru et al. assessed the PLN enlargement by CT or MRI and reported that the PLN enlargement is not an independent risk factors for the OS (HR, 1.514; 95% CI: 0.726–3.237; *p* = 0.2690) and DFS (HR, 1.633; 95% CI: 0.792–3.465; *p* = 0.1848) (29). This study demonstrated that the survival outcome in patients with one or two positive PLNs is consistent with that in patients with negative PLN. In addition, three or more positive PLNs were associated with the inferior DMFS, CSS, and OS. In patients with cervical cancer treated with radiotherapy or CCRT, the number of positive PLN played a vital role in predicting survival, especially the DMFS and CSS.

Although PET-CT is another potential imaging tool to evaluate the involvement of lymph node, PET-CT is not utilized in this study based on following reasons. First, PET-CT is not routinely used for cervical cancer. Only 7 eligible patients performed PET-CT in this study. The number of lymphadenopathy detected by PET-CT is compatible with MRI or CT in these 7 patients. Second, around 85% arise cervical cancer in the less developed regions of the world (1, 30). Clinical examination should be based on their cost-effectiveness and the anticipated expenses for the management. Our finding assumes importance particularly in resource-constrained low-middle-income countries with the highest burden of locally advanced cervical cancer. The present result of a suitable predictor would help identify locally advanced cervical cancer patients who could benefit from definitive CCRT and who might develop distant metastasis after the treatment. For patients at high risk of distant metastasis, adopting radiation therapy with systemic therapy (31), such as adjuvant chemotherapy may help to further improve the treatment outcomes.

This study established that histology was also another prognostic factor for the OS that is compatible with previous studies. Lee et al. conducted a large retrospective study from the Korea National Cancer Incidence Database and reported that the survival was considerably lower in adenocarcinoma than in patients with squamous cell carcinoma in the era of CCRT (HR, 1.4; 95% CI: 1.30–1.50) (32). Moreover, recently, Eriko et al. demonstrated that patients with locally advanced cervical cancer with adenocarcinoma/adenosquamous carcinoma histology experience substantially worse survival outcomes than those with squamous cell carcinoma after CCRT (HR, 1.94; 95% CI: 1.07–3.35) (33). In addition, we established that the use of brachytherapy was the only prognostic factor for the LRFS (HR, 13.15; 95% CI: 2.66–65.10; *p* = 0.002). The American Brachytherapy Society suggested that the EQD2 dose to involve the tumor area was, at least, 80 Gy (34). In the subgroup of patients without brachytherapy, the delivery of boost dose with EBRT was limited because of the tolerance of healthy tissue. The mean dose of EBRT delivered in patients without brachytherapy was 58.6 (range: 50.4–71.6) Gy, which is much lower than the suggested dose and could account for the inferior locoregional control.

This study had some limitations. First, retrospective study carries the unavoidable risk of selection bias. Second, radiographic positive lymph node could not represent the final histopathological result. However, the aim of this study was trying to establish the prognostic value of lymph node numbers detected by MRI and CT in non-surgical group of cervical cancer patients, rather than to reflect the histopathological results.

In conclusion, radiographic numbers of positive PLNs ≥3 was associated with poor survival outcomes and predicted the OS, CSS, and DMFS in patients with cervical cancer that were treated with definitive CCRT or IMRT. Overall, this new category could facilitate better prognostic discrimination of patients with cervical cancer.

## Author contributions

S-CW conceived and designed the study, collected, analyzed, and interpreted the data, prepared the draft and gave final approval of the version to be submitted. L-CL and Y-TK interpreted and undertook data analysis and carried out clinical revision of the data. Y-WL conceived the study, analyzed and interpreted the data, gave final approval of the version to be submitted. All authors read and approved the final manuscript.

### Conflict of interest statement

The authors declare that the research was conducted in the absence of any commercial or financial relationships that could be construed as a potential conflict of interest.
